# Transcriptome Profiling of Etridiazole-Exposed Zebrafish (*Danio rerio*) Embryos Reveals Pathways Associated with Cardiac and Ocular Toxicities

**DOI:** 10.3390/ijms242015067

**Published:** 2023-10-11

**Authors:** Bala Murali Krishna Vasamsetti, Kyongmi Chon, Chang-Young Yoon, Juyeong Kim, Ji-Yeong Choi, Sojeong Hwang, Kyeong-Hun Park

**Affiliations:** Toxicity and Risk Assessment Division, Department of Agro-Food Safety and Crop Protection, National Institute of Agricultural Sciences, Rural Development Administration, Iseo-myeon, Wanju-gun 55365, Jeollabuk-do, Republic of Korea; vbmk84@korea.kr (B.M.K.V.); hsj102@korea.kr (S.H.); blueour@korea.kr (K.-H.P.)

**Keywords:** cardiac toxicity, developmental toxicity, etridiazole, ocular toxicity, pesticide toxicity, transcriptome analysis, zebrafish

## Abstract

Etridiazole (EDZ) is a thiadiazole-containing fungicide commonly used to control *Pythium* and *Phytophthora* spp. Although previous studies have shown that EDZ is teratogenic, the exact molecular mechanisms underlying its toxicity remain unknown. In this study, a zebrafish (*Danio rerio*; ZF) model was used to explore the molecular pathways associated with EDZ toxicity. The whole transcriptome of ZF embryos exposed to 96 h of EDZ was analyzed, along with developmental abnormalities. EDZ-induced malformations were primarily related to the eyes, heart, and growth of the ZF. Compared to untreated ZF, etridiazole-treated ZF had 2882 differentially expressed genes (DEGs), consisting of 1651 downregulated genes and 1231 upregulated genes. Gene ontology enrichment analysis showed that DEGs were involved in biological processes, such as sensory perception, visual perception, sensory organ development, and visual system development, and showed transmembrane transporter and peptidase regulator activities. Metabolism, phototransduction, aminoacyl-tRNA biosynthesis, MAPK signaling pathway, calcium signaling pathway, and vascular smooth muscle contraction were among the most enriched KEGG pathways. The qPCR analyses of the eight random genes were in good agreement with the transcriptome data. These results suggest several putative mechanisms underlying EDZ-induced developmental deformities in ZF.

## 1. Introduction

Thiadiazoles and their derivatives are increasingly used as fungicides worldwide [1,2]. Etridiazole (5-ethoxy-3-trichloromethyl-1,2,4-thiadiazole; EDZ) is a thiadiazole fungicide applied to control *Pythium* and *Phytophthora* spp. [3]. Because EDZ is used for golf course turf, cotton, ornamental plants, citrus, and coffee, runoff from application locations may infiltrate into surface water and harm non-target species [3]. Although data on EDZ distribution in the aquatic environment are limited, the highest reported concentration of 0.23 ngm^−3^ in air (Iowa, USA) and its widespread use could be employed to predict EDZ occurrence in natural waters [4]. According to the general estimated exposure concentration model, the estimated chronic and acute EDZ concentrations caused by turf application in associated water bodies were 32.3 and 230 μg/L, respectively [3]. Because EDZ is not easily biodegradable and is resistant to hydrolysis and aqueous photolysis [3], it may remain in aquatic environments for a long time. With a bioconcentration factor between 93 and 323, the bioconcentration of EDZ in aquatic species is high [5]. EDZ exhibits low acute toxicity in birds, moderate acute toxicity in mammals, acute toxicity in aquatic invertebrates and fish, and high acute toxicity in non-target aquatic plants [3]. According to the available information, degraded 3-dichloromethyl-5-ethoxy-1,2,4-thiadiazole is extremely toxic to aquatic life [3]. The acute toxicity concentration of EDZ to *Daphnia magna* is 3.1 mg/L (48 h EC_50_), while that to *Oncorhynchus mykiss* is 2.4 mg/L (96 h LC_50_), and that to *Danio rerio* is 25.58 mg/L (96 h LC_50_) [6].

EDZ is classified as a Group B2 carcinogen and is probably carcinogenic to humans [3]. EDZ is toxic to animals, and, upon acute exposure, it inhibits the enzyme related to the drug-metabolizing system in the liver of Swiss albino mice [7]. EDZ reduces prenatal survival and body weight while increasing skeletal malformations in rabbit fetuses [3]. Trichloroacetonitrile (an EDZ precursor) induces cardiovascular problems in rats, along with embryonic mortality and fetal weight loss [3,8]. EDZ is a genotoxic and mutagenic in vitro [3].

Its small size, transparent body, high fecundity, affordability, and ease of handling make zebrafish (*Danio rerio*; ZF) a suitable aquatic vertebrate model organism for toxicity research [9]. A recent study reported that EDZ is teratogenic to ZF and causes a range of dose-dependent deformities, including ocular, cardiac, and skeletal defects [10]. It is very likely that exposure to EDZ results in significant changes in the gene expression profile of ZF embryos, reflecting changes in molecular pathways associated with toxicity and developmental deformities. Understanding the toxicity mechanisms of pesticides using traditional methods is time-consuming and less informative [9,10,11,12]. Next-generation sequencing is becoming increasingly popular due to its ability to perform high-throughput analysis of complex biological processes [13], which has successfully identified the molecular signaling pathways underlying pesticide-related developmental toxicity in ZF [14,15].

Considering the efficacy of transcriptome analysis in uncovering detailed molecular changes, performing a comprehensive transcriptome analysis of ZF exposed to EDZ would facilitate identification of the specific genes and molecular pathways regulated by EDZ. Simultaneous phenotypic assessment enables correlating these molecular mechanisms with EDZ-induced developmental deformities. We thus investigated the molecular responses of ZF embryos exposed to EDZ using a high-throughput RNA sequencing technique combined with developmental toxicity assessment. Our results shed light on the concomitantly disrupted signaling pathways that can lead to developmental defects in EDZ-treated ZF.

## 2. Results

### 2.1. Developmental Deformity Assessment

**Figure 1:**
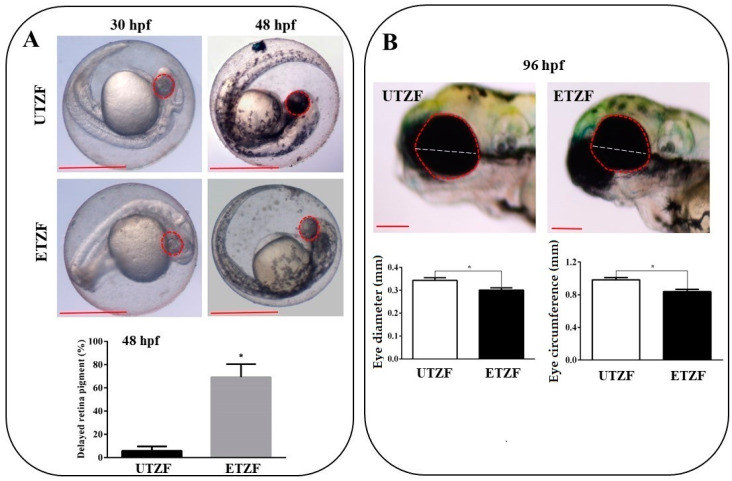
Etridiazole exposure induced ocular toxicity in zebrafish. (**A**) Pictures showing the retinal pigment accumulation at 30 and 48 hpf in untreated (upper panel) and etridiazole-treated (lower panel) zebrafish embryos. Graph showing the percentage of eyes with delayed retina pigmentation. (**B**) Pictures (upper panel) showing eye circumference (red dotted line) and diameter (white dotted line) of untreated and etridiazole-treated zebrafish embryos at 96 hpf. Graphs (lower panels) representing the eye diameter (mm; left graph) and eye circumference (mm; right graph) of the untreated and etridiazole-treated zebrafish embryos. Red dotted circles, eyes; white dotted line, eye diameter; UTZF, untreated zebrafish; ETZF, etridiazole-treated zebrafish. Scale = 0.5 mm. * Statistically significant (*p* < 0.05).

**Figure 2:**
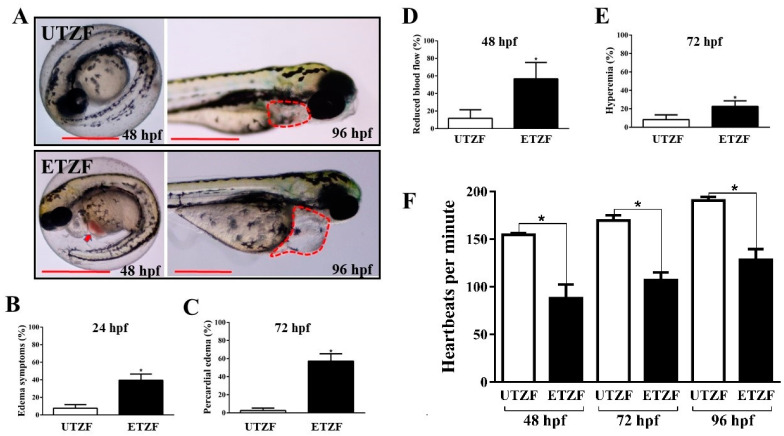
Etridiazole exposure-induced cardiac toxicity in zebrafish embryos/larvae. (**A**) Representative images showing pericardial edema (red dotted lines) and hyperemia (red arrow) in etridiazole-treated zebrafish embryos/larvae. Graphs showing the percentage of (**B**) edema symptoms, (**C**) pericardial edema, (**D**) embryos that showed reduced blood flow, and (**E**) hyperemia observed at designated time points. (**F**) Graph showing average heartbeats per minute scored at 48, 72, and 96 hpf in untreated and etridiazole-treated zebrafish embryos/larvae. The results are presented as mean ± SD. Unpaired Student’s *t*-test was applied for statistical significance evaluation. * Statistically significant (*p* < 0.05). UTZF, untreated zebrafish; ETZF, etridiazole-treated zebrafish. Scale = 0.5 mm.

**Figure 3:**
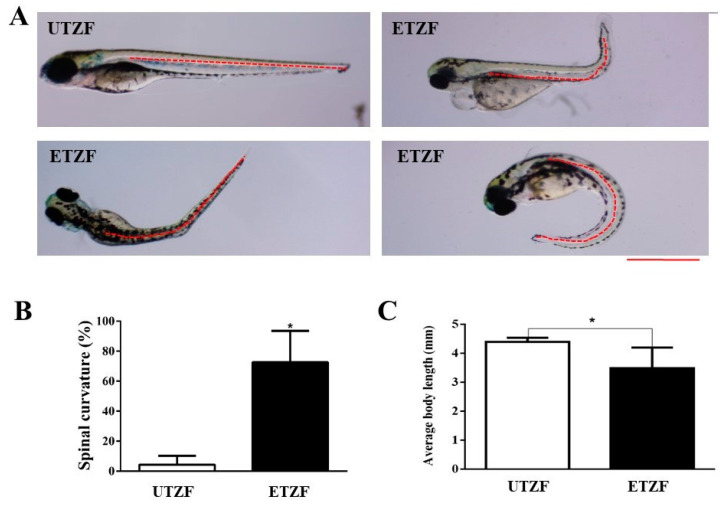
Etridiazole affected the growth and development of zebrafish. (**A**) representative images showing the spine shape of the untreated and etridiazole-treated zebrafish. The red dashed line represents the shape of the spine. (**B**) Bar graph representing the percentage of spinal curves scored at 144 hpf in untreated and etridiazole-treated zebrafish. (**C**) Bar graph representing the average body length of the zebrafish measured at 144 hpf in untreated and etridiazole-treated zebrafish. The results are presented as mean ± SD (*n* = 6). Unpaired Student’s *t*-test was applied for statistical significance evaluation. * Statistically significant (*p* < 0.05). UTZF, untreated zebrafish; ETZF, etridiazole-treated zebrafish. Scale = 0.5 mm.

To assess the toxic effects of EDZ at a test concentration of 20 mg/L [10], multiple morphological deformities of EDZ-exposed ZF were evaluated 24 to 144 h post fertilization (hpf). ZF embryos exposed to E3 buffer served as controls. Toxicity tests demonstrated that EDZ induced a variety of developmental abnormalities in EDZ-treated ZF (ETZF) compared to untreated ZF (UTZF), most of which were related to the eyes, heart, and growth of ZF (Figure 1, Figure 2 and Figure 3).

Several eye-related morphological parameters were evaluated to determine the ocular toxicity of EDZ in ZF. Most ETZF showed no evidence of retinal pigment accumulation at 30 hpf, whereas all UTZF showed slight retinal pigment accumulation (Figure 1A). While more than 95% of UTZF retinas turned black at 48 hpf, only approximately 25% of ETZF had black pigments in their retina, and the remaining 75% of ETZF showed less pigment accumulation than UTZF (Figure 1A). Almost all ETZF (20 mg/L) accumulated retinal pigmentation similar to UTZF by 96 hpf; however, fish treated with 40 mg/L EDZ showed reduced retinal pigmentation (Figure S1). These findings imply that EDZ delays accumulation of retinal pigments in ZF. Additionally, the ETZF group showed significantly smaller eyes than the UTZF group (Figure 1B). Compared with UTZF, ETZF had a 14.76% smaller eye circumference and a 12.54% smaller eye diameter at 96 hpf. The inhibitory effect of EDZ on eye growth was further enhanced by a higher concentration (40 mg/L), with a 50.10% smaller eye circumference and a 48.40% smaller eye diameter in ETZF than in UTZF (Figure S1). These results suggest that EDZ induces ocular toxicity in ZF.

Several well-known indicators of cardiac abnormalities were examined to understand the cardiac toxicity of EDZ in ZF. ETZF displayed aberrant cardiac growth and function, including a high prevalence of pericardial edema (PE), abnormal blood flow, hyperemia, and decreased heart rate (Figure 2), demonstrating that EDZ causes cardiac toxicity in ZF. ETZF showed a high incidence of pericardial edema; at 24 hpf, approximately half of ETZF had edema symptoms (Figure 2B), which increased to 57.02 ± 4.86% by 72 hpf (Figure 2C). Nearly half of the animals treated with EDZ showed abnormal blood flow (Figure 2D), which led to hyperemia in ETZF (Figure 2E). The ETZF group exhibited a significantly lower heart rate than the UTZF group (Figure 2F). The UTZF group mean heart rate was 154.8 ± 1.7 (48 hpf), 169.8 ± 5.4 (72 hpf), and 190.8 ± 3.7 (96 hpf) bpm, whereas it was 88.0 ± 14.6 (48 hpf), 107.0 ± 8.3 (72 hpf), and 128.4 ± 11.3 (96 hpf) bpm in the ETZF group.

ETZF showed a significantly higher spinal curvature than UTZF, including scoliosis, lordosis, and kyphosis (Figure 3A). Spinal curvatures accounted for 4.3 ± 5.9% in UTZF, and 72.6 ± 21.0% in ETZF (Figure 3B). EDZ treatment significantly influenced the growth of ZF (Figure 3C). At 144 hpf, the average body length of UTZF and ETZF was 4.4 ± 0.1 and 3.5 ± 0.6 mm, respectively. These findings suggest that EDZ influences the growth and development of ZF.

### 2.2. Whole Transcriptome Assessment

**Figure 4:**
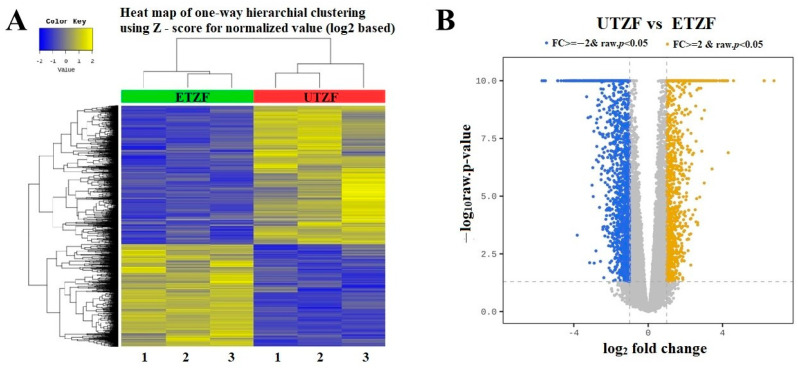
(**A**) Heat map of differential expression between ETZF and UTZF. (**B**) Volcano plot for differentially expressed genes (DEGs) of UTZF vs. ETZF. Downregulated (FC ≤ −2, and FDR < 0.05) genes and upregulated (FC ≥ 2, and FDR < 0.05) genes are shown in blue and yellow, respectively.

To understand the molecular signatures underpinning EDZ-induced developmental deformities, the transcriptomes of UTZF and ETZF were probed using high-throughput whole-transcriptome analysis. Illumina-based sequencing generated an average of 65,195,447, and 74,131,946, total reads for UTZF and ETZF, respectively (Table 1). Among them, an average of 64,122,571 (85.3%) and 72,831,267 (83.15%) clean reads were filtered for UTZF and ETZF. More than 98% of clean reads fulfilled the Q20 requirement, whereas more than 95% met the Q30 criterion. On average, 84.23% of clean reads were successfully mapped to the reference genome (GRCz11). A heatmap of the one-way hierarchical clustering analysis using the z-score computed from the normalized value (log2-based) is shown in Figure 4A. To highlight the overall distribution of transcripts and identify differentially expressed genes (DEGs) after EDZ exposure, a volcano plot was constructed using the fold change of each transcript and *p*-value (Figure 4B). In ETZF, 2882 genes were differentially expressed (|FC| ≥ 2 & raw. *p* < 0.05) compared to UTZF (Figure S2). Of these, 1231 genes were upregulated, and 1651 genes were downregulated. A list of all genes that showed up- and downregulation in response to EDZ is shown in Supplementary data 1. The top upregulated (110 to 15 fold) genes after exposure to EDZ included heat shock cognate 70-kd protein, like (*hsp70l*), heat shock cognate 70-kd protein, tandem duplicate 1, transcript variant X2 (*hsp70.1*), heat shock cognate 70-kd protein, tandem duplicate 3 (*hsp70.3*), chemokine (C-X-C motif) ligand 18b (*cxcl18b*), matrix metallopeptidase 13a b (*mmp13a*), podocan-like protein 1 (*LOC101882092*), FOS-like antigen 1a (*fosl1a*), ISG15 ubiquitin-like modifier (*isg15*), B-cell CLL/lymphoma 3 (*bcl3*), tumor necrosis factor, alpha-induced protein 2b (*tnfaip2b*), and heat shock protein 90, alpha (cytosolic), class A member 1, tandem duplicate 2 (*hsp90aa1.2*). The top downregulated genes (-50 to -15 fold) in EDZF were crystallin beta gamma X (*crybgx)*, zonadhesin, transcript variant X1 (*LOC100147904*), crystallin, alpha A (*cryaa*), cytochrome P450, family 7, subfamily A, polypeptide 1 (*cyp7a1*), crystallin, gamma M2d11 (*crygm2d11*), crystallin, gamma M2d13 (*crygm2d13*), phosphorylase kinase, alpha 1b (muscle), transcript variant X1 (*phka1b*), opsin 1 (cone pigments), medium-wave-sensitive, 1 (*opn1mw1*), crystallin, gamma M2d17 (*crygm2d17*), crystallin, gamma M2d15 (*crygm2d15*), crystallin, gamma M2d8 (*crygm2d8*), opsin 1 (cone pigments), short-wave-sensitive 1 (*opn1sw1*), crystallin, gamma M2d9 (*crygm2d9*), crystallin, gamma M2d5 (*crygm2d5*), crystallin, gamma M2d7 (*crygm2d7*), crystallin, gamma M2d20 (*crygm2d20*), crystallin, gamma M2d16 (*crygm2d16)*, Rho guanine nucleotide exchange factor (GEF) 15 (*arhgef15*), crystallin, beta A2b (*cryba2b*), crystallin, gamma M2d14 (*crygm2d14*), crystallin, gamma M2d10 (*crygm2d10*), crystallin, gamma M2d21 (*crygm2d21*), retinitis pigmentosa 1-like 1b, transcript variant X1 (*rp1l1b*), crystallin, gamma M2d18 (*crygm2d18*), crystallin, gamma MX (*crygmx*), crystallin, gamma M2d19 (*crygm2d19*), and guanine nucleotide binding protein (G protein), gamma transducing activity polypeptide 1 (*gngt1*).

**Figure 5:**
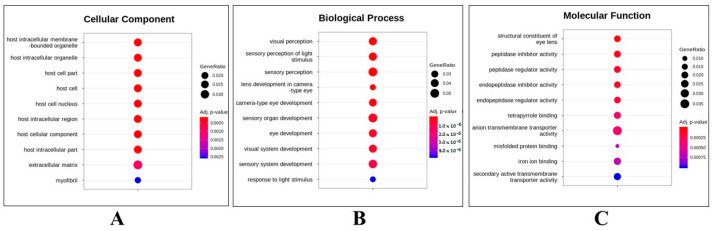
Top-ten terms obtained in gene ontology functional analysis. Cellular component (CC) (**A**), biological process (BP) (**B**), and molecular function (MF) (**C**) of differentially expressed genes in ZF after EDZ exposure.

To understand the molecular mechanisms that affect EDZ exposure, gene ontology (GO) over-representation analysis of statistically significant DEG events between UTZF and ETZF was carried out, dividing DEGs into cellular components (CC), biological processes (BP), and molecular functions (MF; Figure 5). Most of the DEGs belonged to the host intracellular region and extracellular matrix (Figure 5A) and were involved in biological processes related to eye development and function (Figure 5B). Molecular functions, such as peptidase inhibitor activity, peptidase regulator activity, anion transmembrane transporter activity, and iron-binding activities, were influenced by EDZ in ZF (Figure 5C).

**Figure 6:**
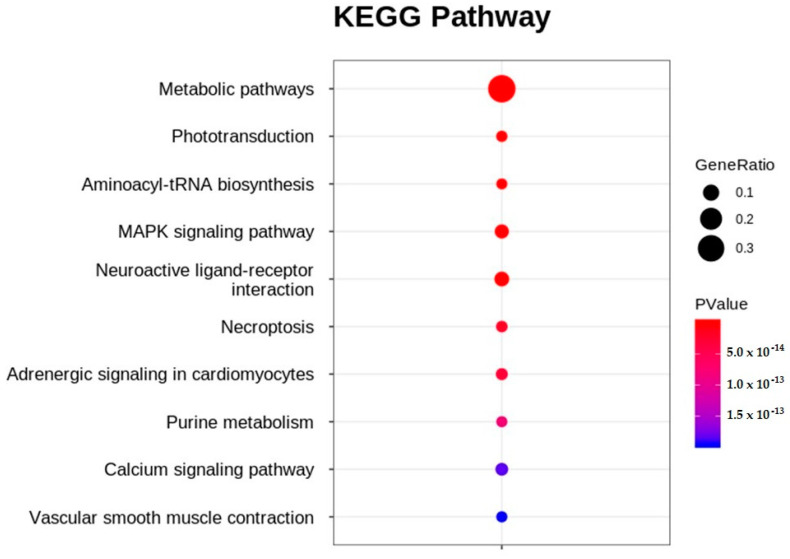
Top-ten enriched KEGG pathways obtained in etridiazole-treated, compared to untreated, zebrafish.

Significant DEGs were analyzed using Kyoto Encyclopedia of Genes and Genomes (KEGG) pathway enrichment analysis. When the *p*-value was adjusted to ≤0.05, 91 pathways were enriched in the KEGG pathway analysis (Figure S3). Of these, approximately 50% (45 pathways) were related to metabolism, followed by cellular processes (14 pathways), organismal systems (14 pathways), environmental information processing (13 pathways), genetic information processing (3 pathways), and human diseases (2 pathways). The topten enriched KEGG pathways included metabolic pathways, phototransduction, aminoacyl-tRNA biosynthesis, the MAPK signaling pathway, neuroactive ligand-receptor interaction, necroptosis, adrenergic signaling in cardiomyocytes, purine metabolism, the calcium signaling pathway, and vascular smooth muscle contraction (Figure 6). Table 2 lists the genes that showed up- and downregulation tendencies in the top-ten enriched KEGG pathways.

To verify the accuracy of the transcriptome sequencing data, eight randomly selected DEGs that showed up- or downregulated expression in ETZF compared to that in UTZF were quantified by qPCR. The qPCR results for the tested genes showed an expression trend comparable to that of the RNA sequencing data (Table 3), thus confirming the accuracy of the transcriptome data.

## 3. Discussion

Pesticides have complex chemical profiles and are known to interfere with several biological functions in organisms [16], making it difficult and time-consuming to unravel the molecular mechanisms of off-target pesticide toxicity using standard approaches. Next-generation high-throughput sequencing technology, on the other hand, has been successful in demonstrating pesticide toxicity at the molecular level in off-targets, particularly ZF [14,15]. Therefore, we employed transcriptome analysis, in combination with developmental toxicity assessment, to understand the molecular mechanisms associated with EDZ toxicity in the ZF model. The transcriptome analysis revealed 2882 significant DEGs, including 1231 upregulated and 1651 downregulated genes, and revealed the functional characteristics of the gene sets in the earlier stages of ZF development in response to EDZ exposure. This study identified molecular signatures of EDZ toxicity that could be used to elucidate the mechanisms underlying cardiac and ocular toxicity.

Fish are susceptible to ocular toxicity because they reside in water that exposes their eyes to pollutants [17]. Pollutants damage the retina in various ways, including morphological changes, molecular damage, and irregularities in electrical signaling and phototransduction [17]. Several results obtained in this study support the notion that EDZ induces ocular toxicity in ZF. First, the results of the morphological assessment after EDZ exposure (20 mg/L) showed that EDZ suppressed eye development and retinal pigment accumulation (Figure 1). Of note, these ocular abnormalities were significantly exacerbated with increased EDZ (40 mg/L; Figure S1), supporting the hypothesis that EDZ affects ocular development, and, consequently, vision, in ZF. Indeed, other studies have also shown that EDZ delays retinal pigment accumulation in ZF in a dose-dependent manner [10], and retinal hypopigmentation has been associated with vision issues in ZF [18]. Interestingly, functional analysis of significant DEGs of ETZF results were in line with the ocular deformity data. The results showed that the biological processes critical for the development and perception of the visual system, such as eye development, sensory perception of light stimulus, visual perception, camera-type eye development, lens development in camera-type eye, visual system development, and response to light stimulus, were among the top GO terms (Figure 5), indicating that EDZ exerts its toxicity on the development and function of the visual system of ZF via several molecular mechanisms. In addition, phototransduction, a signaling pathway essential to vision, as it converts light information into electrical impulses that can be understood by the brain [19], was one of the ten most enriched signaling pathways in the KEGG analysis (Figure 6), suggesting visual abnormalities in ETZF.

Thirty-one genes associated with the phototransduction pathway were significantly downregulated in ETZF and consisted of opsins (*opn1mw1* and *opn1mw2*), recoverins (*rcvrn3*, *rcvrn2*, and *rcvrna*), guanine nucleotide-binding proteins (*gnat 1* and *gnat2*), and guanylate cyclase-activating proteins (*guca1c*, *guca 1b*, *guca1d*, *guca1e*, and *guca1g*) (GCAPs) (Table 2). Opsins are important for detecting light signals and converting them into electrical signals that the brain can understand [20]. The opsin genes *opn1mw1* and *opn1mw2* encode medium-wavelength-sensitive cone opsins that sense light in the green wavelength range, and mutations or abnormalities in these genes result in ZF with reduced color vision or color discrimination [21]. Recoverins govern phototransduction termination in mammalian rods. Zhang et al. have shown that recoverin degradation increases the recovery of cone photoresponse in ZF [22]. G protein subunit alpha transducin 1 (Gnat1) participates in the visual transduction cascade in the retina and is necessary for rod photoreceptor development in ZF [23]. GCAPs are mostly found in the rod and cone photoreceptor cells of the retina, where they control the activity of guanylate cyclase by detecting changes in intracellular Ca^2+^ levels during illumination [24]. Guanylate cyclase 2F, retinal (*Gucy2f*)-knockout ZF larvae exhibited visual impairment and photoreceptor layer dystrophy, as well as the loss and shortening of the outer segments of cones and rods [25]. Phosphodiesterase 6 B (*Pde6b*) knockdown causes an abnormal photoreceptor cell shape and reduces visual function in ZF embryos [26]. Guanylate cyclase activator 1B (GUCA1B) is expressed in photoreceptor cells, and its overexpression increased cGMP levels, improving visual performance [27]. Grk1b and Grk7a both contributed to the restoration of the isolated cone photoresponse in larval ZF [28]. Mutations in GRK1A can disrupt normal rhodopsin regulation and impair the activity of the phototransduction pathway, and, thus, vision, in ZF [29]. The top upregulated gene following EDZ exposure, the heat shock cognate 70-kd protein, like (*hsp70l*), was reported to be involved in embryonic lens formation [30]. Therefore, it is highly likely that EDZ causes ocular toxicity by altering the expression of several key genes involved in eye development and function of ZF. However, the precise roles of these genes in ZF ocular toxicity require further investigation.

Key signs of ZF cardiac development and functional abnormalities, including pericardial edema, circulatory disorders, and bradycardia, were observed in ETZF (Figure 2), consistent with the observations of Vasamsetti et al. [10]. Moreover, three signaling pathways known to be associated with cardiac function, namely adrenergic signaling in cardiomyocytes (ASC) [31], vascular smooth muscle contraction (VSMC) [32], and calcium signaling (CS) [33], were among the top-ten enriched KEGG signaling pathways (Figure 5). Furthermore, the MAPK pathway, which plays a crucial role in the development and regeneration of the ZF heart [34], was among the top-ten enriched KEGG pathways, suggesting that exposure to EDZ in the early developmental stages leads to cardiac developmental and functional problems in ZF, involving the disruption of numerous molecular signaling pathways.

The calcium signaling pathway Is critical for multiple biological signaling pathways [35,36] and is essential in heart development and function [37]. Cardiomyocyte contractility is critically dependent on proper maintenance of intracellular Ca^2+^ homeostasis, and Ca^2+^ homeostasis anomalies can contribute to contractile dysfunction and arrhythmias in various clinical scenarios, including heart failure [38]. Cardiogenesis requires a several-fold increase in cytoplasmic Ca^2+^ in the cardiac region [39]; therefore, disruptions in calcium signaling prevent proper cardiogenesis and, consequently, cardiac function. Therefore, the low cardiac output observed in the ETZF group (Figure 2) may have been caused by abnormalities in the calcium signaling pathway. EDZ downregulated several key genes involved in calcium signaling (Table 2), and the importance of some of these genes in ZF heart development and function is discussed. Myosin light chain kinase 4B (MYLK4B) disruption results in improper heart function and circulation, as well as aberrant blood vessel development in ZF [40]. Calcium/calmodulin-dependent protein kinase (CaMK) is a protein kinase family that plays important functions in calcium signaling and the regulation of numerous physiological processes, including excitation and contraction coupling [41,42]. Adrenoreceptors are G protein-coupled receptors (GPCRs) that are ubiquitously expressed and mediate the cellular effects of adrenaline and noradrenaline [43] and were downregulated (*adrb2b*, *adra1bb*, and *adra1d*) in ETZF (Table 2). According to a previous study, β-adrenergic receptors play an essential role in the modulation of heart function in ZF during development, with β-1AR and β-2bAR being most closely linked to heart rate regulation [44]. Endothelin receptor type B (EDNRB) is a GPCR that regulates a variety of physiological functions, including cardiovascular development, and is known to cause abnormal ventricular looping and lower blood flow in ZF [45].

The main function of highly specialized vascular smooth muscle cells (VSMC) is to control blood flow and pressure by contracting the blood vessel and reducing its diameter [32]. The expression of myosin light chin-related kinases (MLCKs) was downregulated in ETZF (*mylk4b*, *mylk4a*, and *mylk2*; Table 2). MLCKs regulate smooth muscle contraction in ZF by phosphorylating the myosin light chain (MLC), and its knockout in ZF embryos results in cardiac function and circulation abnormalities [46].

ASC is a process in which norepinephrine (NE) is released in cardiomyocytes, leading to the activation of β1-adrenergic receptors and the production of cyclic AMP (cAMP) [47], which has been shown to be involved in metabolic reprogramming, causing myocardial cells to switch to glycolysis and divide [48]. Several genes associated with this signaling pathway, such as calcium voltage-gated channel subunits (*cacng6b*, *cacna1sb*, and *cacng8b*), ATPase sodium potassium transporters (*atp1b2b*, *atp1a3b*, and *atp1b2a*), and adrenergic receptors (*adrb2b*, *adra1bb*, and *adra1d*) were downregulated in ETZF (Table 2). In vertebrates, myocardial contraction rate and force are maintained by adrenergic receptors (ARs), and ARs have been found to be implicated in heart rate control in ZF. ZF larvae, with the deletion for both *2aAR* and *2bAR*, had higher heart rates than controls, but those without a single AR expression exhibited lower heart rates [44].

Given that protein synthesis components, such as tRNAs and aminoacyl synthases, were found to be downregulated in ZF that displayed growth retardation following pesticide treatment in earlier studies [14,15], protein biosynthesis disruption may explain the growth retardation observed in ETZF (Figure 3). Notably, aminoacyl-tRNA biosynthesis, a critical mechanism that connects amino acids to their corresponding transfer RNA (tRNA) molecules to ensure proper protein synthesis, was one of the ten most enriched KEGG pathways in ETZF (Figure 6), suggesting abnormal protein synthesis in ETZF. The biosynthesis of 15 tRNAs (tRNA-histidine, -aspartic acid, -lysine, -glutamic acid, -asparagine, -tyrosine, -glycine, -isoleucine, -glutamine, -proline, -arginine, -cysteine, -threonine, and -phenylalanine) was downregulated following EDZ exposure in ZF (Table 2). In addition to their best-known function of transporting amino acids during protein synthesis, recent research has shown that tRNAs are necessary for transcriptional and translational regulation, post-translational modification, stress, and diseases [49]. Revendro et al. observed that decreased embryo survival and higher malformations were caused by tRNA dysregulation in ZF and hypothesized that this promotes protein aggregation and chromosomal instability [49]. According to Waldrone et al., inhibiting histidyl-tRNA synthetase in ZF promotes cell cycle arrest and cell death in neural progenitor cells in vivo [50]. The results of Mullen et al. support the idea that histidyl-tRNA synthetase mutations promote dysregulated protein synthesis in neurons [51]. Further evidence from the current research that supports the notion that EDZ may disrupt protein synthesis in ZF is the enrichment of MAPK signaling in the KEGG pathway analysis (Figure 6). MAPK signaling enzymes play a significant role in the regulation of nucleosome and gene expression, mRNA stability, and translation [52,53,54]. MAPK signaling pathways are necessary for converting extracellular impulses into intracellular impulses to control cell proliferation, differentiation, survival, tissue regeneration, and organogenesis throughout the ZF life cycle [53,54,55]. Certainly, more research is needed to fully appreciate the hypothesis that the disruption of protein synthesis is one of the causes of EDZ toxicity in ZF.

The EDZ concentration used in this study corresponded to the average EC_50_ of EDZ in ZF at 96 hpf [10], ensuring that most abnormal phenotypes induced by EDZ were included in the transcriptome sample. This consequently offered us the possibility to directly correlate the molecular changes revealed in the transcriptome analysis with the prevalent EDZ-induced abnormalities, particularly those associated with ocular and cardiac deformities. While this approach allowed us to gain valuable insights into the potential molecular mechanisms underlying EDZ-induced toxicity, it is important to acknowledge the limitations associated with the chosen concentration. The concentration used in this study may not accurately reflect real-world exposure scenarios. The use of a high concentration also entails the potential of non-specific effects and activation of stress response pathways that may be irrelevant at lower, environmentally realistic concentrations, potentially leading to an overestimation of pathways associated with EDZ toxicity. Consequently, the results should be interpreted with caution, and follow-up studies should examine the effects of EDZ at lower, more environmentally relevant concentrations to provide a more comprehensive understanding of its toxicological profile.

## 4. Materials and Methods

### 4.1. Ethics Statement

All animal experiments were performed following the standards of care and use of laboratory animals and approved by the Animal Ethics Committee of the National Institute of Agricultural Sciences, Rural Development Administration, Republic of Korea (NAS-202102).

### 4.2. Mating and Embryo Collection

ZF (AB strain) were cultured as previously described [15]. One day before the start of the developmental toxicity assay, well-fed males and females (1:1 ratio) were mated at 27 °C for embryo collection. Embryo collection and washing were performed as previously described [10,15].

### 4.3. Etridiazole Exposure and Assessment of Developmental Deformities

The test concentration of EDZ (20 mg/L) corresponds to the average EC_50_ value of EDZ against ZF at 96 hpf [10]. Etridiazole (99.6%, Sigma-Aldrich, St. Louis, MO, USA) solution was dissolved in E3 buffer. Fifty to sixty embryos (2.0–3.0 hpf) were dropped into petri dishes containing either 20 mg/L EDZ or E3 buffer. The developmental toxicity test was carried out using a total of six 24-well plates. For each test condition, 20 embryos were placed in each 24-well plate. The embryos/larvae were anesthetized with ethyl 3-aminobenzoate methanesulfonate (0.1%; Sigma, St. Louis, MO, USA) as needed.

### 4.4. Cardiac Abnormality Analyses

A heartbeat study was performed as described previously [11]. Three time intervals (48, 72, and 96 hpf) were selected for the heartbeat study. Embryos and larvae were anesthetized and placed under a microscope (Stemi 508, Zeiss, Oberkochen, BW, Germany) to expose the heart beats. Each embryo or larva’s heartbeat was counted for 20 s, and the results were translated to beats per minute. Blood flow in the dorsal artery and hyperemia were observed under a microscope (Stemi 508; Zeiss, Oberkochen, BW, Germany) at 48 hpf. Embryos exhibiting slower, discontinuous, or no blood flow compared to the normal blood circulation observed in the untreated control group were categorized as having “abnormal blood flow”. Five embryos from each 24-well plate were scored under each experimental condition [n = 6 (24-well plates), 30 embryos].

### 4.5. Eye size Measurement

The eye diameter and circumference were measured at 96 hpf. ZF larvae were anesthetized, and eye images were obtained using a stereomicroscope (Stemi 508, Zeiss, Oberkochen, BW, Germany). The circumference and diameter of the eyes were measured using the OptiView 3.7 software (Korealabtech, Seongnam, Republic of Korea). Ten larvae from each 24-well plate were used for measurements under each test condition [n = 6 (24-well plate), 60 embryos].

### 4.6. Body Length Survey

ZF larvae (144 hpf) were anesthetized to estimate body length, and images were taken under a stereomicroscope at 144 hpf. Body lengths were obtained using the OptiView 3.7 software (Korealabtech, Seongnam, Republic of Korea). Five embryos were screened from each 24-well plate under each test condition [n = 6 (24 well plate), 30 embryos].

### 4.7. Statistical Analysis

Developmental deformities data were collected from a total of six 24-well plates, and the percentage of each abnormal phenotype observed in UTZF and ETZF was calculated for each individual 24-well plate. Mean, standard deviation, and normality (Kolmogorov-Smirnov and Shapiro-Wilk) of the obtained data were assessed (Prism 5.0; GraphPad, San Diego, CA, USA). The statistical significance of the developmental deformity data was assessed using an unpaired t-test (Prism 5.0; GraphPad, San Diego, CA, USA). Statistical significance was set at *p* < 0.05.

### 4.8. Pair-end RNA-Sequencing and DEG Selection

For transcriptome analysis, three samples containing 30–40 embryos/larvae under each test condition were frozen (liquid nitrogen) and stored. The experimental protocols for RNA extraction, quantification, and cDNA library preparation were based on our previous studies [14,15]. Paired-end sequencing (2 × 100 bp) was carried out on the NovaSeq platform (Illumina, Inc., San Diego, CA, USA).

Differentially expressed genes (DEGs) between ETZF and UTZF were filtered using DESeq2 and nbinomWaldTest. For significant DEG selection, a cut-off of the absolute log2-fold change of 2 and a *p*-value of 0.05 were set as criteria.

### 4.9. DEG Analysis

The significant DEGs obtained following EDZ exposure were analyzed using the Gene Ontology (GO) g: Profiler tool (https://biit.cs.ut.ee/gprofiler/, accessed on 24 February 2023) and KEGG pathway enrichment analyses (http://www.kegg.jp/kegg/pathway.html; accessed on 25 February 2023).

### 4.10. qPCR

Three samples of UTZF and ETZF, each containing 30-40 embryos/larvae, were used for qPCR-based validation of the transcriptome data. The experimental techniques for RNA extraction, cDNA preparation, and qPCR were based on our previous study [14]. Under each test condition, RNA was extracted from three biological replicates, and 1000 ng of RNA was used for cDNA conversion following the manufacturer’s protocol (ReverTra Ace™ qPCR RT master mix with gDNA remover (Toyobo, Osaka, Japan)). Before cDNA conversion, RNA samples were subjected to DNase I treatment. For the qPCR experiment, 20 nanograms of cDNA were used as a template. qPCR amplification and SYBR detection were performed using the CFX96 Dx real-time PCR detection device from Bio-rad, CA, USA. Each sample was tested with three technical replicates, and a reaction mixture containing nuclease-free water was used as a negative control. TOPreal^TM^ qPCR 2X premix from Enzynomics, Daejon, Republic of Korea, was employed for this analysis. For the qPCR experiment using the 2^−ddCT^ approach, the fold expression was analyzed. The oligonucleotide primers used for the qPCR are shown in Table S1.

## 5. Conclusions

As EDZ is commonly used in agriculture, it is critical to comprehend its toxicity in aquatic species. The current study is the first in-depth investigation of the transcriptomes of EDZ-exposed ZF. The results showed that EDZ induces a variety of anomalies, most notably those affecting the eyes, heart, and the growth and development of ZF. In addition, transcriptome results showed that EDZ alters the expression of a myriad of genes and exerts its ocular and cardiac toxicity through multiple molecular mechanisms. These results not only shed light on the molecular signaling pathways that are altered in response to EDZ and lead to developmental ZF damage, but also help to better elucidate the mechanisms of cardiac and ocular toxicity in other organisms. Toxicity screening procedures utilized in ZF, in conjunction with transcriptome analysis, enable the detection of off-target effects and the investigation of putative underlying mechanisms at a lower cost than those involved in animal investigations. Given the high degree of genetic homology, understanding the pathways triggered by pesticide poisoning in ZF could provide clues regarding analogous responses in other animals and humans [56].

## Figures and Tables

**Table 1:** Summary of transcriptome sequence data showing sequence qualities and mapping.

Samples	Total Reads	Processed Reads	Q20 (%)	Q30 (%)	GC (%)	Mapped Reads	Unmapped Reads
UTZF-1	72,783,012	71,783,716	98.91	96.05	43.62	60,458,452 (84.22%)	11,325,264 (15.78%)
UTZF-2	61,925,392	60,857,682	98.91	96.06	44.95	51,896,692 (85.28%)	8,960,990 (14.72%)
UTZF-3	60,878,020	59,726,314	98.85	95.87	46.00	51,603,485 (86.40%)	8,122,829 (13.60%)
ETZF-1	88,823,364	87,431,000	99.06	96.55	50.43	70,539,606 (80.68%)	16,891,394 (19.32%)
ETZF-2	67,022,082	65,997,010	99.88	96.00	46.40	55,889,349 (84.68%)	10,107,661 (15.32%)
ETZF-3	66,550,392	65,065,792	98.97	96.27	47.32	55,066,792 (84.10%)	10,414,184 (15.90)

UTZF, untreated zebrafish; ETZF, etridiazole-treated zebrafish; Q20 (%), percentage of Phred quality score 20 percentage; Q30 (%), percentage of Phred quality score 30; GC (%), percentage of guanine–cytosine.

**Table 2:** List of differently expressed genes of top-ten enriched KEGG pathways.

KEGG Pathway	Differentially Expressed Genes	*p*-Value	False Discovery Rate	Bonferroni
**Metabolic** **pathways**	*cyp7a1*, *si:ch73-111m19.2*, *LOC559107*, *tdo2a*, *gda*, *cyp2r1*, *gls2a*, *pde6c*, *bco1l*, *cyp27b1*, *elovl4b*, *cyp8b1*, *nme2a*, *agxta*, *chia.2*, *zgc:77938*, *pde6b*, *ATP8*, *gyg1b*, *si:ch211-135f11.4*, *arg1*, *gc3*, *cyp2p9*, *alas2*, *gucy2f*, *tecra*, *si:ch211-217a12.1*, *sdsl*, *zgc:103586*, *hsd20b2*, *pnp4b*, *hacd1*, *agxtb*, *pde6g*, *zgc:153896*, *rdh8b*, *ND3*, *si:ch211-264e16.1*, *cyp2p8*, *si:dkey-91i10.3*, *si:ch211-214p16.3*, *spam1*, *ND4L*, *tecrl2b*, *nt5e*, *si:ch211-132f19.7*, *cyp2p6*, *anpepb*, *dpm3*, *ugt5d1*, *ugt1b5*, *LOC100331665*, *zgc:92162*, *zgc:153704*, *si*, *gpx8*, *ATP6*, *nt5c1aa*, *pde6a*, *chs1*, *ND1*, *dgat2*, *cyp2p7*, *hyal6*, *si:ch211-127i16.2*, *ugt5b3*, *hao2*, *si:dkey-73n8.3*, *mdh1ab*, *aanat1*, *g6pca.2*, *acer1*, *cyp27a1.4*, *ND2*, *cel.2*, *pfkma*, *atpv0e2*, *entpd8*, *fam213b*, *cel.1*, *csgalnact1b*, *dgat1a*, *cahz*, *ugt1b2*, *agpat3*, *haao*, *lpin1*, *pcxb*, *gls2b*, *elovl8a*, *gpat3*, *hsd11b1la*, *sephs1*, *guk1b*, *pisd*, *ptgdsb.2*, *sgms2*, *etnppl*, *dhrs3a*, *ndufa4*, *si:dkey-78a14.4*, *acot12*, *upp1*, *tecrl2a*, *ND5*, *ahcyl1*, *LOC101886891*, *COX3*, *ugt5g1*, *ckmt2a*, *ND4*, *ND6*, *gbgt1l2*, *gstt2*, *ugt5b4*, *tdh2*, *ca2*, *adss*, *cyp27a1.2*, *suv39h1a*, *LOC100536156*, *si:ch73-55i23.1*, *si:ch211-197l9.4*, *si:ch73-71d17.1*, *dpys*, *ptges3a*, *got2a*, *zgc:153031*, *b3gnt5a*, *mocs1*, *ndufa11*, *pde5aa*, *entpd5a*, *elovl2*, *amd1*, *fech*, *atp5g3a*, *st3gal7*, *pnp6*, *th2*, *gstk1*, *entpd3*, *ugt1a4*, *gal3st1a*, *cds1*, *pdha1b*, *alg10*, *ndufab1b*, *mcee*, *hpda*, *nme6*, *gucy1a3*, *CYTB*, *si:ch211-256m1.8*, *ca12*, *alpl*, *si:dkey-103j14.5*, *gc2*, *pcyt1bb*, *pde6d*, *uqcr10*, ***mars*****, *atic*, *cbsb*, *LOC100333801*, *adprm*, *cbr1l*, *eno4*, *sat1a.2*, *nnt2*, *ak7b*, *ckmb*, *gnsb*, *pnp5b*, *LOC103911725*, *Glyctk*, *tcirg1b*, *shmt2*, *lss*, *galm*, *ugt2a6*, *paics*, *gnpda2*, *ptgis*, *renbp*, *si:ch211-276a23.5*, *got1l1*, *gsr*, *phgdh*, *ckma*, *gsto2*, *pla2g4aa*, *hk2*, *hyi*, *zgc:163121*, *ugt2a4*, *acsl4b*, *abat*, *hao1*, *taldo1*, *sqrdl*, *cyp51*, *galns*, *gfpt1*, *acsl2*, *odc1*, *amdhd2*, *adi1*, *scd*, *gpx1b*, *gpx3*, *psat1*, *eprs*, *si:dkey-175m17.6*, *ethe1*, *mthfr*, *pycr1b*, *p4ha2*, *pfkfb3*, *tph1b*, *adh8a*, *tdo2b*, *cmbl*, *bhmt*, *rdh12l*, *ptgs2b*, *mthfd1l*, *gstp1*, *si:ch211-199m3.4*, *dao.1*, *hmox1a*, *sqlea*, *ptgs2a*, *gldc*, *cox4i1l*, *p4ha1b*, *cyp24a1*, *gstp2***	1.47446 × 10^−90^	2.24117 × 10^−88^	2.24117 × 10^−88^
**Phototransduction**	*opn1mw1*, *gngt1*, *rho*, *rhol*, *guca1c*, *guca1d*, *grk1b*, *gnat2*, *grk7a*, *guca1e*, *opn1mw2*, *guca1g*, *pde6b*, *gc3*, *guca1b*, *gucy2f*, *rcvrn3*, *cnga1*, *si:dkey-22i16.2*, *rgs9a*, *slc24a1*, *pde6g*, *pde6a*, *grk1a*, *calm3a*, *saga*, *calm1b*, *rcvrna*, *rcvrn2*, *gc2*	4.87074 × 10^−27^	7.40352 × 10^−25^	3.70176 × 10^−25^
**Aminoacyl-tRNA** **biosynthesis**	*trnH*, *trnD*, *trnS2*, *trnK*, *trnL2*, *trnE*, *trnN*, *trnY*, *trnG*, *trnI*, *trnQ*, *trnP*, *trnM*, *trnR*, *trnC*, *trnT*, *trnL1*, *trnF*, ***farsa*****, *mars*, *dars*, *gars*, *nars*, *vars*, *yars*, *aars*, *iars*, *eprs*, *tars***	7.95807 × 10^−22^	1.20963 × 10^−19^	4.03209 × 10^−20^
**MAPK** **signaling**	*hsc70*, *ppm1na*, *fgf1a*, *tgfa*, *cacng6b*, *ppm1nb*, *fgf4*, *dusp10*, *nr4a1*, *dusp2*, *si:ch73-55i23.1*, *mapkapk2b*, *ppp3r1a*, *cacna1sb*, *egf*, *fgf1b*, *cacng8b*, *LOC795766*, *fgf20a*, ***tgfbr1b*****, *fosab*, *csf1ra*, *flna*, *insrb*, *met*, *taok3a*, *fgfr1b*, *gadd45bb*, *map3k14a*, *tnfrsf1a*, *irak4*, *kitlgb*, *pla2g4aa*, *tab1*, *gadd45aa*, *prkacba*, *nfkb1*, *angpt2a*, *mapk12b*, *gadd45ba*, *epha2a*, *rasgrp3*, *jun*, *ddit3*, *pgfa*, *gadd45ga*, *si:dkey-4p15.3*, *fas*, *mknk2b*, *hspb1*, *il1b*, *hsp70.3*, *hsp70.1*, *hsp70l***	3.48302 × 10^−19^	5.29419 × 10^−17^	1.32355 × 10^−17^
**Neuroactive** **ligand-receptor interaction**	*gabra6a*, *drd4b*, *sstr2b*, *si:dkey-1h24.2*, *gabrr1*, *LOC100007535*, *try*, *mc1r*, *lpar4*, *mtnr1ab*, *prlra*, *glrba*, *ednrba*, *si:dkey-202l22.3*, *adrb2b*, *chrna3*, *lpar6b*, *gabra6b*, *edn3b*, *prlhr2a*, *ptger1a*, *adra1bb*, *LOC100332187*, *gabrz*, *LOC556532*, *apln*, *p2ry1*, *chrna2b*, *htr1ab*, *adra1d*, *LOC100330650*, *Chrne*, *agtr2*, *vipr2*, *oxtrl*, *glra3*, ***galn*****, *ltb4r2a*, *npb*, *tbxa2r*, *p2ry6*, *LOC567978*, *Agt*, *htr1fb*, *lepa*, *c5*, *lepr*, *chrna5*, *c3a.3*, *chrng*, *sst1.2*, *f2r*, *pomca*, *ptger2b*, *f2rl1.2*, *ltb4r2b*, *rln1*, *c3a.6*, *c3a.1*, *adma***	9.66551 × 10^−17^	1.46916 × 10^−14^	2.93831 × 10^−15^
**Necroptosis**	*usp21*, *slc25a6*, *slc25a4*, *si:dkeyp-50d11.2*, *tlr4ba*, *camk2d1*, *si:ch73-55i23.1*, *h2afx*, ***stat1a*****, *tnfrsf1a*, *jak1*, *casp8*, *caspa*, *pla2g4aa*, *tnfaip3*, *irf9*, *LOC100006428*, *zgc:194125*, *zgc:92066*, *zgc:92480*, *si:ch211-202f3.3*, *hsp90aa1.1*, *zgc:198419*, *zgc:109934*, *stat3*, *zgc:173593*, *zgc:173594*, *fas*, *LOC558816*, *stat1b*, *sqstm1*, *il1b*, *hsp90aa1.2***	1.80786 × 10^−14^	2.74794 × 10^−12^	4.5799 × 10^−13^
**Adregenic** **signaling in cardiomycetes**	*atp1b2b*, *cacng6b*, *si:ch211-132f19.7*, *scn4bb*, *zgc:163073*, *atp1a3b*, *adrb2b*, *calm3a*, *pln*, *scn4ba*, *slc9a1*, *myl2b*, *si:ch211-225p5.8*, *atp1b2a*, *adra1bb*, *camk2d1*, *calm1b*, *adra1d*, *cacna1sb*, *cacng8b*, *agtr2*, *tnnt2d*, ***creb5a*****, *creb3l3a*, *cmlc1*, *cremb*, *creb3l3l*, *atp1a1a.2*, *agt*, *acta1a*, *prkacba*, *tpma*, *mapk12b*, *actc1b*, *tnnt2c*, *pik3r5*,**	2.96038 × 10^−14^	4.49978 × 10^−12^	6.42826 × 10^−13^
**Purine** **metabolism**	*Gda*, *pde6c*, *nme2a*, *pde6b*, *gc3*, *gucy2f*, *pnp4b*, *pde6g*, *nt5e*, *si:ch211-132f19.7*, *nt5c1aa*, *pde6a*, *entpd8*, *guk1b*, *adss*, *LOC100536156*, *pde5aa*, *entpd5a*, *pnp6*, *entpd3*, *nme6*, *gucy1a3*, *gc2*, *pde6d*, ***atic*****, *adprm*, *ak7b*, *pnp5b*, *paics*, *si:ch211-199m3.4***	7.85124 × 10^−14^	1.19339 × 10^−11^	1.49174 × 10^−12^
**Calcium** **signaling** **pathway**	*camk1ga*, *si:rp71-17i16.4*, *phkg1a*, *mylk4b*, *stim2a*, *mylk4a*, *si:dkey-1h24.2*, *fgf1a*, *camk1gb*, *mylk2*, *si:ch211-132f19.7*, *slc25a6*, *fgf4*, *phkg1b*, *slc25a4*, *ednrba*, *adrb2b*, *calm3a*, *pln*, *ptger1a*, *adra1bb*, *LOC101886891*, *camk2d1*, *camk4*, *calm1b*, *ppp3r1a*, *adra1d*, *cacna1sb*, *egf*, *fgf1b*, *fgf20a*, *oxtrl*, ***ltb4r2a*****, *met*, *fgfr1b*, *mst1ra*, *mst1*, *tbxa2r*, *prkacba*, *mcoln3a*, *f2r*, *ltb4r2b*, *cxcr4a***	1.84649 × 10^−13^	2.80666 × 10^−11^	2.98174 × 10^−12^
**Vascular smooth muscle contraction**	*mylk4b*, *mylk4a*, *mylk2*, *si:ch211-132f19.7*, *zgc:92162*, *LOC101885790*, *calm3a*, *ppp1r14aa*, *edn3b*, *adra1bb*, *acta2*, *calm1b*, *si:ch73-55i23.1*, *prkg1b*, *adra1d*, *cald1b*, *cacna1sb*, *myh11a*, *gucy1a3*, *ramp2*, ***nppa*****, *prkg1a*, *LOC567978*, *agt*, *pla2g4aa*, *prkacba*, *myh9a*, *arhgef1b*, *si:ch73-265d7.2*, *adma***	1.96167 × 10^−13^	2.98174 ×0^−11^	2.98174 × 10^−12^

Upregulated genes (≥2 fold) are shown in bold text.

**Table 3:** qPCR comparison of randomly selected up- and downregulated DEGs from transcriptome data.

Gene Symbol	Fold Change (RNA-Seq)	Fold Change (qPCR) (Mean ± SD)
*hsp70l*	109.43	189.03 ± 71.21
*fosl1a*	9.10	7.54 ± 3.04
*cbx7a*	9.10	11.82 ± 6.30
*atf3*	7.84	8.99 ± 4.76
*mir181b-1*	−5.86	−10.11 ± 4.98
*alas2*	−4.28	−17.52 ± 18.21
*cyldb1*	−3.26	−8.19 ± 2.58
*rhcga*	−8.08	−22.69 ± 8.91

## Data Availability

Data are contained within the article or Supplementary Materials.

## Supplementary Materials

The following supporting information can be downloaded from https://www.mdpi.com/article/10.3390/ijms242015067/s1.
