# Supplementary material for: Transcriptome Profiling of Etridiazole-Exposed Zebrafish (Danio rerio) Embryos Reveals Pathways Associated with Cardiac and Ocular Toxicities
Source: Int J Mol Sci. 2023 Oct 11;24(20):15067. doi: 10.3390/ijms242015067 (PMC10606920; doi:10.3390/ijms242015067)
Supplement: Supplementary file 1 [file ijms-24-15067-s001.zip › Supplementary Table 1.docx]

**Supplementary Table S1.** List of oligo primers used in qPCR validation

| **Gene**  **(Sequence ID)** | **Orientation** | **Oligo sequence** |
| --- | --- | --- |
| *hsp70l*  (NM_001113589) | Forward | CGACCTCTTCAGGGGAACACTA |
|  | Reverse | TCTGAGCCTTGTCCATCTTGG |
| *fosl1a*  (NM_001161552) | Forward | CTGTGGCAGGATCAGGTCAG |
|  | Reverse | AAGTGCCCTTGATGGTCCTG |
| *cbx7a*  (NM_001017853) | Forward | GCCTGAGACCTCGTCAAGTT |
|  | Reverse | GTTTTGGTCCACTTCGGTGC |
| *atf3*  (NM_200964) | Forward | AAGCGAGAGGTAGTTTGGCT |
|  | Reverse | GGTCGTTCTCCTCTGGGACA |
| *mir181b-1*  (NR_029925) | Forward | CATTCATTGCTGTCGGTGGG |
|  | Reverse | ATCTGGGCCACAGTTACATTCA |
| *alas2*  *(*NM_131682) | Forward | TCCACTGACCCCCAAAATCG |
|  | Reverse | CGTGGACCTCATCCACGAAA |
| *cyldb*  *(*XM_009306058) | Forward | GACTGACCGTGTATGGGGTG |
|  | Reverse | CCCAACGTCCCATCTGAACA |
| *rhcga*  *(*NM_001089577) | Forward | GGAGCTCTGCTGGGAAAAGT |
|  | Reverse | TCCTCGACCGCATAGAGAGT |
