# Supplementary material for: Transcriptome Profiling of Etridiazole-Exposed Zebrafish (Danio rerio) Embryos Reveals Pathways Associated with Cardiac and Ocular Toxicities
Source: Int J Mol Sci. 2023 Oct 11;24(20):15067. doi: 10.3390/ijms242015067 (PMC10606920; doi:10.3390/ijms242015067)
Supplement: Supplementary file 1 [file ijms-24-15067-s001.zip › Supplementory file .pdf]

Transcriptome Profiling of Etridiazole-Exposed Zebrafish (*Danio rerio*) Embryos Reveals Pathways Associated with Cardiac and Ocular Toxicities

Bala Murali Krishna Vasamsetti [1], Kyongmi Chon [1,*] Chang-Young Yoon[1], Juyeong Kim[1], Ji-Yeong Choi[1], Sajeong Hawng [1], and Kyeong-Hun Park[1]

[1]Toxicity and Risk Assessment Division, Department of Agro-Food Safety and Crop Protection, National Institute of Agricultural Sciences, Rural Development Administration, Wanju-gun 55365, Republic of Korea

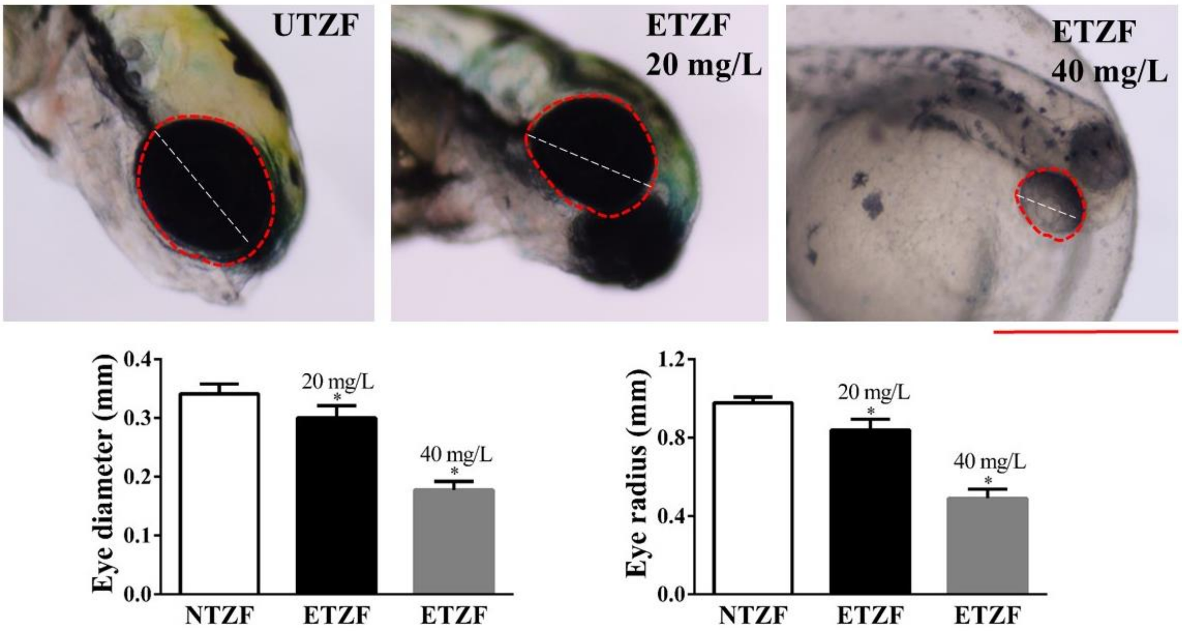

Figure S1. Etridiazole exposure induced ocular toxicity in zebrafish. Pictures showing retinal pigment accumulation and eye size at 96 hpf in the UTZF and ETZF. Graphs show the percentage of the eye diameter (mm) (left graph) and eye circumference (mm) (right graph) of the UTZF and ETZF. Red dotted circles: eyes; white dotted line: eye diameter; UTZF: untreated zebrafish; ETZF: etridiazole-treated zebrafish. Scale = 0.5mm.

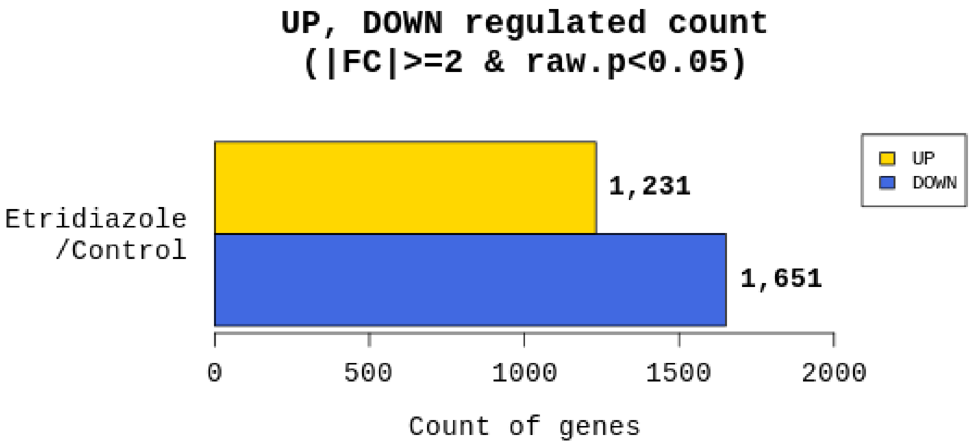

Figure S2. Images showing the number of up- and down- regulated DEGs after etridiazole exposure.

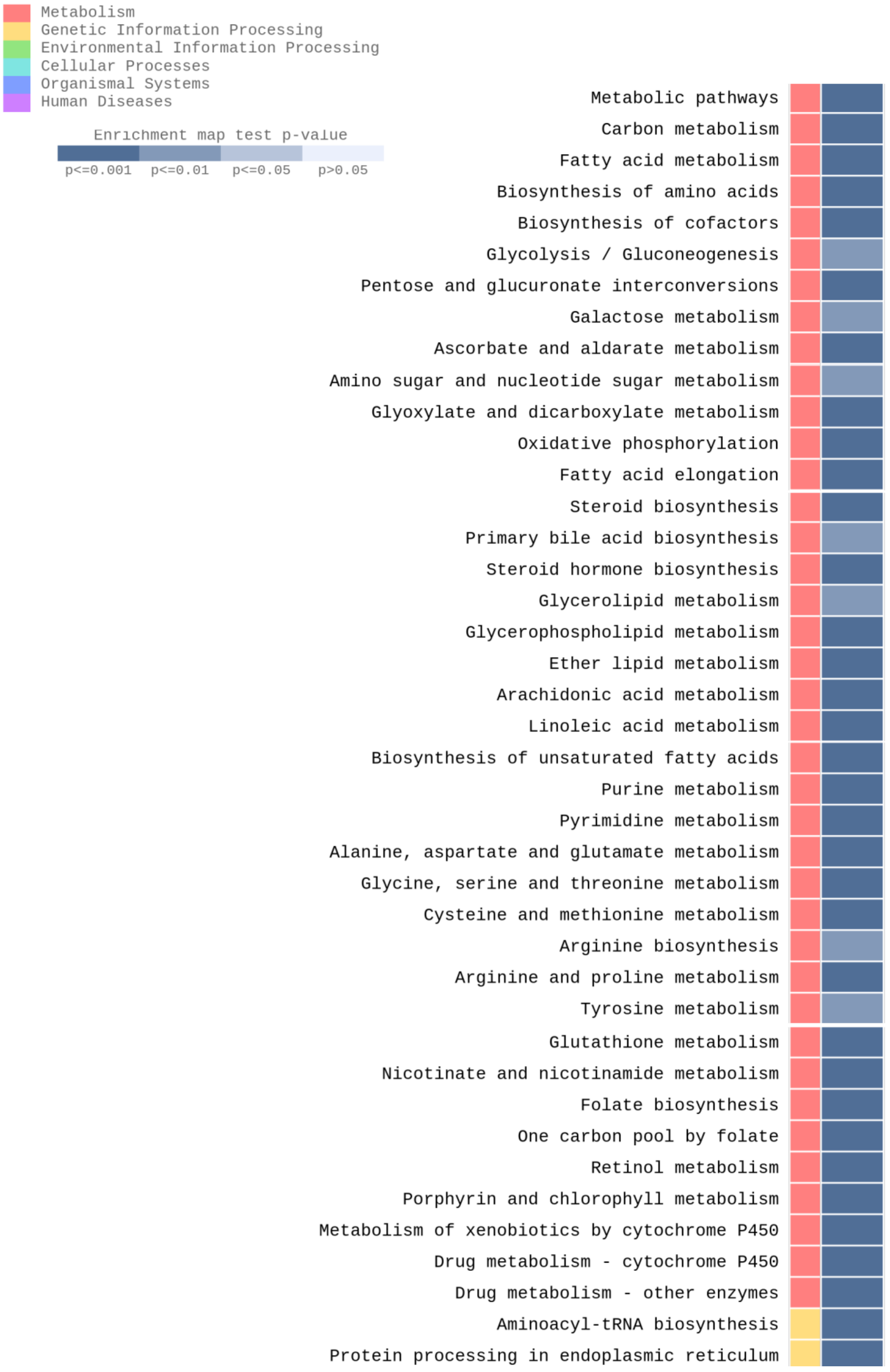
Metabolism
Genetic Information Processing
Environmental Information Processing
Cellular Processes
Organismal Systems
Human Diseases
Enrichment map test p-value
p<=0.001
p<=0.01
p<=0.05
p>0.05
Metabolic pathways
Carbon metabolism
Fatty acid metabolism
Biosynthesis of amino acids
Biosynthesis of cofactors
Glycolysis / Gluconeogenesis
Pentose and glucuronate interconversions
Galactose metabolism
Ascorbate and aldarate metabolism
Amino sugar and nucleotide sugar metabolism
Glyoxylate and dicarboxylate metabolism
Oxidative phosphorylation
Fatty acid elongation
Steroid biosynthesis
Primary bile acid biosynthesis
Steroid hormone biosynthesis
Glycerolipid metabolism
Glycerophospholipid metabolism
Ether lipid metabolism
Arachidonic acid metabolism
Linoleic acid metabolism
Biosynthesis of unsaturated fatty acids
Purine metabolism
Pyrimidine metabolism
Alanine, aspartate and glutamate metabolism
Glycine, serine and threonine metabolism
Cysteine and methionine metabolism
Arginine biosynthesis
Arginine and proline metabolism
Tyrosine metabolism
Glutathione metabolism
Nicotinate and nicotinamide metabolism
Folate biosynthesis
One carbon pool by folate
Retinol metabolism
Porphyrin and chlorophyll metabolism
Metabolism of xenobiotics by cytochrome P450
Drug metabolism - cytochrome P450
Drug metabolism - other enzymes
Aminoacyl-tRNA biosynthesis
Protein processing in endoplasmic reticulum

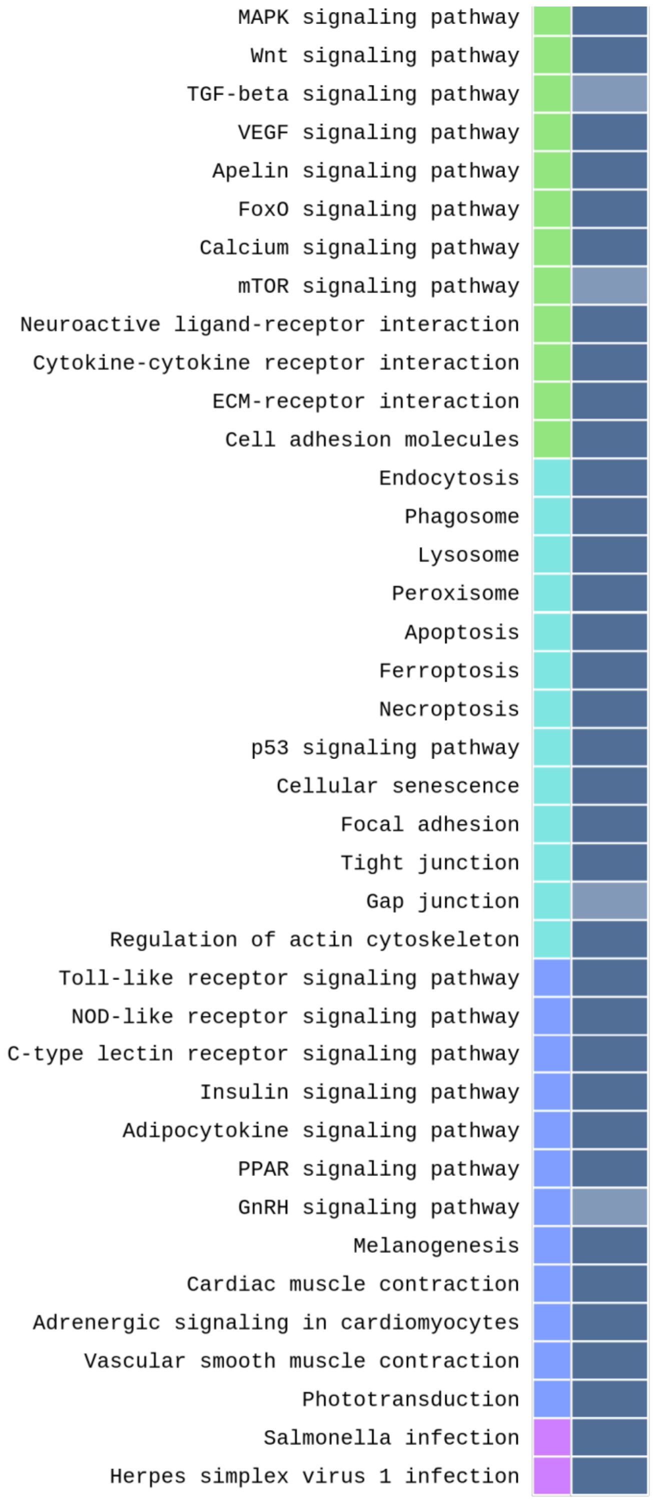

Figure S3. Graph showing the list of enriched KEGG pathways after etridiazole treatment.
